# Association between anthropometric measures and cardiovascular disease (CVD) risk factors in Hainan centenarians: investigation based on the Centenarian’s health study

**DOI:** 10.1186/s12872-018-0810-8

**Published:** 2018-05-02

**Authors:** Qiao Zhu, Xiao-Bing Wang, Yao Yao, Chao-Xue Ning, Xiao-Ping Chen, Fu-Xin Luan, Ya-Li Zhao

**Affiliations:** 1 Hainan Branch of PLA General Hospital, Sanya, 572000 China; 2 Department of Military Education and Training, Naval Aeronautical and Astronautical University, Yantai, 264001 China

**Keywords:** Centenarians, BMI, WC, WHR, WHtR, CVD risk factorts

## Abstract

**Background:**

Centenarians refer to a special group who have outlived most of their fellows. Body shape and abdominal obesity have been identified as cardiovascular disease (CVD) risk factors. Our study aimed to evaluate the relationship between body mass index (BMI), waist circumference (WC), waist-to-hip ratio (WHR), and waist-to-height ratio (WHtR) and CVD risk factors among male and female centenarians in Hainan province.

**Methods:**

Five hundred thirty-seven centenarians aged between 100 and 115 (Mage = 107 years old) years participated in this study. Each participant received a standardized questionnaire and physical examination. We measured anthropometric variables (BMI, WC, WHR, WHtR, SBP and DBP) and serum lipid (TC, TG, HDL-C and LDL-C).

**Results:**

76.9% (*n* = 413) of the study subjects were female. TC, TG, LDL-C and HDL-C were significantly higher in female group than that of male group. BMI, WC and WHtR were well-correlated with the CVD risk factors. The anthropometric measures were negatively related with HDL-C levels and positively related with the other CVD risk factors.

**Conclusions:**

Hainan centenarians were short in stature and underweight. Moreover, female centenarians were often pear-shaped, while male centenarians were often apple-shaped. Further, BMI, WC and WHtR were well-correlated with the serum lipid, and TC, TG, LDL-C and HDL-C were significantly higher in females than males. Also, BMI, WC and WHtR were closely related to the incidence of dyslipidemia in females, including high TG, high LDL-C and low HDL-C.

## Background

Centenarians represent a special group that deserves more attention. They have outlived most of their fellows. Data, anthropometric measures and biochemical indicators, from centenarians can be collected and used to study. So, our study aimed to elucidate the relationship between anthropometric measurements and cardiovascular disease (CVD) risk factors in Hainan centenarians.

## Methods

### Study population

Five hundred thirty-seven centenarians (413 female and 124 male) were enrolled in the study between June 2014 and October 2016 in Hainan. All participants provided written informed consent. The study protocol was approved by the ethical committee of the Hainan branch of PLA General Hospital (Sanya, China).

### Anthropometric measurements

Anthropometric indices, Height (H), Weight (W), waist circumference (WC) and hip circumference (HC), were measured with participants dressed in light clothing and barefoot. We measured the height and weight of the elderly with a scale (seca, Germany). Each parameter was measured twice. We computed the BMI, WHR and WHtR using the following standard formula: BMI=W/H^2^, WHR = WC/HC, WHtR = WC/H.

### Serum analysis

Blood samples were analysed by the Hainan Branch of PLA General Hospital (Sanya, China). TC, TG, LDL-C and HDL-C were measured by electrochemiluminescence (Roche automatic biochemical analyser, cobas 6000, USA).

### Blood pressure measurement

Two blood pressure recordings were measured by electronic sphygmomanometers (Omron HEM-7200, Japan). If the difference between the first and second measurement was more than 5 mmHg, the repeated measurements were performed.

### Definition of CVD risk factors

Hypertension was defined as having one or more of the following: (1) a systolic BP ≥140 mmHg, (2) a diastolic BP ≥ 90 mmHg, (3) physician-diagnosed hypertension. Dyslipidemia was defined according to the Chinese guidelines on the prevention and treatment of dyslipidemia in adults (2007): TC ≥ 240 mg/dl as high; LDL-C ≥ 160 mg/dl as high; HDL-C < 40 mg/dl as low; and TG ≥ 200 mg/dl as high.

### Statistical analysis

We used EpiData 3.0 software to double-enter the data, and SPSS 19.0 software (SPSS Inc., Chicago, IL, USA) was used for statistical analysis. Continuous variables were tested for normality using the Kolmogorov-Smirnov test, and chisquare test for categorical variables. Comparisons between males and females were performed using two independent samples t-test or the Mann-Whitney U test for continuous variables. The associations between anthropometric measures and CVD risk factors were firstly examined using Spearman’s correlation coefficients. Receiver Operating Characteristic (ROC) analyses were then used to calculate the area under the ROC curves (AUC). Multiple logistic regressions were used in female centenarians at last. *p* < 0.05 indicated statistical significance.

## Results

### Characteristics of study subjects

We collected 537 centenarians’ personal information (Table 1). There were a statistically significant difference in illiteracy, widowed, alcohol and tea drinking between male and female centenarians. Anthropometric measurements and CVD risk factors are shown in Table 2. Differences on BMI and WHtR were significant. With regards to serum lipids, TC, TG, LDL-C and HDL-C were significantly higher in female group than that of male group. Moreover, the difference was statistically significant in SBP between men and women. Correspondingly, the prevalence of hypertension and high TG were significantly higher in females than males.

**Table 1:** General Information of Study Subjects

Variables	Total (*N* = 537)	Male (*N* = 124)	Female (*N* = 413)	*P*.Value
Age	102.6 ± 2.8	102.3 ± 2.4	102.8 ± 2.9	0.060
Illiterate	87.3	58.1	95.2	<0.001
Widowed	88.8	62.9	93.5	<0.001
Han nationality	83.6	80.6	84.5	0.309
Smoker	9.9	5.6	6.1	0.048
Alcohol drinker	13.6	25.8	9.9	<0.001
Tea drinker	16.6	29.8	12.4	<0.001
No. of children	4.2 ± 2.2	4.5 ± 2.0	4.1 ± 2.3	0.027

*P*-value from Mann-Whitney U test for age and No. of children. Chisquare test for all other categorical variables. These tests were done to compare between males and females

Mean ± standard deviation presented for continuous variables

**Table 2:** Characteristics of Anthropometric Measurements and CVD Risk Factors

Characteristics	Total (*n* = 537)	Male (*n* = 124)	Female (*n* = 413)	*P*-value
Anthropometric measures				
Height (m)	146.00 ± 9.37	155.29 ± 7.60	143.21 ± 7.94	< 0.001
Weight (kg)	38.45 ± 8.72	45.79 ± 7.10	36.24 ± 7.92	< 0.001
Waist (cm)	75.49 ± 9.17	76.60 ± 8.82	75.15 ± 9.26	0.202
Hip (cm)	83.96 ± 7.05	85.93 ± 7.04	83.37 ± 6.96	< 0.001
Anthropometric indices				
BMI	17.99 ± 3.50	18.99 ± 2.66	17.69 ± 3.67	< 0.001
WHR	0.90 ± 0.09	0.89 ± 0.07	0.90 ± 0.09	0.241
WHtR	0.52 ± 0.07	0.49 ± 0.06	0.53 ± 0.07	< 0.001
CVD Risk Factors measurements				
SBP (mm Hg)	153.20 ± 23.99	148.93 ± 21.35	154.48 ± 24.61	0.024
DBP (mm Hg)	74.71 ± 12.72	73.50 ± 11.78	75.07 ± 12.98	0.230
TC (mg/dl)	135.05 ± 28.36	169.70 ± 38.27	185.89 ± 37.49	< 0.001
TG (mg/dl)	103.73 ± 57.92	94.11 ± 41.12	106.63 ± 61.84	0.012
HDL-C (mg/dl)	55.36 ± 14.72	50.88 ± 13.83	56.71 ± 14.73	< 0.001
LDL-C (mg/dl)	109.82 ± 30.80	101.87 ± 29.62	112.21 ± 30.79	0.001
Prevalence of CVD Risk Factors, n (%)				
Hypertension	164.58 ± 17.48	160.44 ± 14.62	181.74 ± 12.71	0.006
High TC	265.78 ± 22.17	263.56 ± 20.50	266.27 ± 22.80	0.774
High TG	280.73 ± 147.83	220.03 ± 17.92	294.22 ± 160.87	0.035
Low HDL-C	34.92 ± 4.20	34.46 ± 4.31	35.26 ± 4.14	0.432
High LDL-C	179.71 ± 20.20	175.43 ± 13.98	180.52 ± 21.25	0.579

*P*-value from two independent samples t-test for WHR, Diastolic Blood Pressure, Hypertension, High Total Cholesterol, High Triglycerides, Low HDL-C and High LDL-C. Mann-Whitney U test for all other continuous variables. These tests were done to compare between males and females

Mean ± standard deviation presented continuous variables

*CVD RF* Cardiovascular disease risk factors

### Correlations between anthropometric measures and CVD risk factor variables

The gender-stratified Spearman’s coefficients for the correlations between the various anthropometric measures and CVD risk factors are shown in Table 3. BMI, WC and WHtR were well-correlated with the various CVD risk factors. The anthropometric measures were negatively related with HDL-C levels and positively related with the other CVD risk factors. However, the relationship is not fairly distributed between women and men.

**Table 3:** Spearman Correlation Coefficient between Anthropometric Indices and Cardiovascular Disease Risk Factors

Variables	BMI	WC	WHR	WHtR
Overall (*n* = 537)				
SBP	0.115**	0.124**	0.070	0.114**
DBP	0.082	0.139**	0.035	0.095*
TC	0.097*	0.108*	0.058	0.138**
TG	0.219**	0.178**	0.032	0.171**
HDL-C	−0.166**	−0.123**	−0.023	−0.044
LDL-C	0.160**	0.160**	0.075	0.161**
Male (124)				
SBP	0.055	0.170	0.073	0.164
DBP	−0.023	0.228*	0.063	0.147
TC	0.122	0.040	−0.030	0.118
TG	0.268**	0.179*	0.033	0.195*
HDL-C	−0.156	−0.075	− 0.094	−0.015
LDL-C	0.188*	0.101	−0.017	0.137
Female (413)				
SBP	0.162**	0.120*	0.068	0.079
DBP	0.122*	0.119*	0.029	0.074
TC	0.131**	0.151**	0.082	0.108*
TG	0.226**	0.183**	0.022	0.142**
HDL-C	−0.132**	−0.126*	−0.014	−0.104*
LDL-C	0.188**	0.194**	0.097*	0.141**

The associations between anthropometric measures and CVD risk factors were examined using Spearman’s correlation coefficients

** All are significant at the level of< 0.01(2-tailed)

* All are significant at the level of< 0.05(2-tailed)

### Association of various anthropometric measures and CVD risk factors using ROC curve analyses

The area under the ROC curves (AUCs) for the association between CVD risk factors and anthropometric measures are shown in Table 4. BMI and WC were associated with the highest AUCs for two of the five CVD risk factors (high TG and low HDL-C), and WHtR was associated with the highest AUC for high TG. However, the differences in the AUCs for the various anthropometric measures were often small, demonstrating overlapping 95% confidence intervals (CIs).

**Table 4:** Adjusted Area Under Receiver Operating Characteristic (ROC) Curve for the Various Anthropometric Indices and Cardiovascular Disease Risk Factors

Variables	BMI	WC	WHR	WHtR
Hypertension	0.546 (0.492-0.600)	0.551 (0.496-0.605)	0.536 (0.481-0.590)	0.531 (0.478-0.585)
High TC	0.530 (0.448-0.611)	0.548 (0.457-0.639)	0.529 (0.433-0.626)	0.527 (0.434-0.620)
High TG	**0.698 (0.591-0.805)**	**0.638 (0.519-0.757)**	0.475 (0.365-0.586)	**0.631 (0.520-0.742)**
Low HDL-C	**0.661 (0.595-0.728)**	**0.578 (0.502-0.654)**	0.536 (0.463-0.609)	0.513 (0.438-0.588)
High LDL-C	0.577 (0.499-0.654)	0.574 (0.482-0.666)	0.516 (0.420-0.611)	0.554 (0.459-0.649)

Anthropometric measure with the highest AUC value in **bold**

### Association of various anthropometric indices and CVD risk factors in female centenarians

Multiple logistic regressions were used to evaluate the association between anthropometric indices and CVD risk factors in female centenarians, as shown in Table 5. The incidence of high TG, low HDL-C and high LDL-C were closely related to BMI, 10.59 times, 2.30 times and 2.16 times, respectively. Besides, high TG is also associated with WC (4.24 times unadjusted and 3.66 times adjusted). Furthermore, low HDL is associated with WHtR (2.87 times unadjusted and 3.11 times adjusted). However, it should be noted that there were a very few centenarians with high TG with WHR ≤0.8. So, the data in this column showed infinity.

**Table 5:** Multiple Logistic Regression between Anthropometric Indices and CVD Risk Factors

Variables	BMI		WC		WHR		WHtR	
	(>17.5 VS ≤ 17.5)		(>80 VS ≤ 80)		(>0.8 VS ≤ 0.8)		(>0.6 VS ≤ 0.6)	
	Unadjusted	Adjusted	Unadjusted	Adjusted	Unadjusted	Adjusted	Unadjusted	Adjusted
Hypertension	1.07 (0.69~ 1.65)	1.09 (0.69~ 1.72)	1.04 (0.63~ 1.73)	0.92 (0.54~ 1.57)	1.05 (0.49~ 2.27)	1.04 (0.47~ 2.29)	1.42 (0.6~ 3.21)	1.57 (0.66~ 3.71)
High TC	1.24 (0.60~ 2.55)	0.38 (0.10~ 1.48)	2.31* (1.10~ 4.87)	1.70 (0.49~ 5.95)	0.89 (0.26~ 3.07)	1.09 (0.14~ 8.72)	1.51 (0.50~ 4.56)	0.67 (0.10~ 4.58)
High TG^a^	10.59** (2.40~ 46.67)	8.95** (2.00~ 40.04)	4.24** (1.62~ 11.05)	3.66* (1.33~ 10.06)	**–**	**–**	3.13 (0.98~ 10.07)	2.62 (0.76~ 9.01)
Low HDL-C	2.30* (1.18~ 4.48)	2.16* (1.08~ 4.30)	1.95 (0.99~ 3.85)	2.02 (0.99~ 4.11)	0.84 (0.28~ 2.51)	0.81 (0.27~ 2.45)	2.87* (1.21~ 6.78)	3.11* (1.26~ 7.68)
High LDL-C	2.16* (1.03~ 4.54)	4.44* (1.10~ 17.93)	2.31* (1.10~ 4.87)	1.45 (0.41~ 5.16)	0.89 (0.26~ 3.07)	0.82 (0.10~ 6.79)	2.02 (0.73~ 5.60)	2.67 (0.44~ 16.19)

Factors in Female Centenarians

The prevalence of hypertension, high level of TC, TG, LDL-C and low level of HDL-C were demonstrated according to anthropometric indices (BMI, WC, WHR, WHtR)

The cut-off points for dyslipidemia were plasma TC ≥ 240 mg/dl and/or use of medications to lower blood cholesterol for high TC, TG ≥ 200 mg/dl for high TG, HDL-C<40 mg/dl for low HDL-C, and LDL-C ≥ 160 mg/dl and/or use of medications to lower blood cholesterol for high LDL-C

According to the level of anthropometric indices, two-class classification was used for grouping (BMI (>17.5 VS ≤ 17.5), WC (>80 VS ≤ 80), WHR (>0.8 VS ≤ 0.8), WHtR (>0.6 VS ≤ 0.6))

The odds ratios were presented as unadjusted and further adjusted for age, illiterate, windowed, Han nationality, smoker, alcohol drinker, tea drinker and No. of children

^a^A very few centenarian with concentration of TG higher than 200 mg/dl in WHR ≤ 0.8 group thus the data showed infinity in this column

*P < 0.05, **P < 0.01

## Discussion

Cardiovascular diseases (CVD) have become serious causes of death among old people. Dyslipidemia is one of the most important independent risk factors for CVD [1, 2]. Increasing the awareness and management of patients with dyslipidemia has a positive impact on CVD prevention.

This study was conducted in Hainan to comprehensive investigate the physical condition of the centenarians as well as the associated CVD risk factors. Our results indicated that the difference was statistically significant in TC, TG, LDL-C and HDL-C between males and females. It was also revealed that BMI, WC and WHtR were negatively related with HDL-C levels and positively related with the other CVD risk factors. Furthermore, our results suggested that the presence of dyslipidemia was significantly associated with body size.

Study has shown that the longevity of people has its own characteristics, such as short stature, light weight and more. Consistent with prior studies, our results showed that Hainan centenarians were short in stature (female 143.21±±7.94 cm, male 155.29 ± 7.60 cm), underweight (female 36.24 ± 7.92 kg, male 45.79 ± 7.10 kg), and BMI (17.99 ± 3.50) was far lower than the cut-off values for Asian populations.

Generally, abdominal fat was positively correlated with cardiovascular risk factors. Much research has been proved that the people in the Asian-pacific regions are easy to gain the abdominal obesity. In the study, we observed that female centenarians were often pear-shaped (WHR ≥ 0.8 for women), while males were often apple-shaped (WHR < 0.9 for men). Our results were in line with previous studies [3, 4]. It has been proposed that circulating gonadal steroids determine these sex-specific differences in adipose tissue distribution, which can be observed even after menopause [5]. Moreover, women’s adipose tissue is more favourable to accumulate in the peripheral and gluteofemoral depots to prevent the development of atherosclerosis [6–8]. In a word, the fat distribution pattern of women contributes to more healthier metabolic status, and may even take part in the determination of the overall life expectancy.

On the other hand, studies reported that age was one of major risk factors of dyslipidemia, which may be related to hereditary characteristics and degenerative processes as well as adipose tissue distribution and progressive development of insulin resistance [9, 10]. In our study, BMI, WC and WHtR were well-correlated with the serum lipid, and TC, TG, LDL-C and HDL-C were significantly higher in females than males. This may be associated with changes in postmenopausal hormone levels [11]. oestrogen levels are lower, glucose and lipid metabolism may differ significantly [12, 13] . In addition, we observed that BMI and WC were exhibited the relationship with high TG and low HDL-C. This was consistent with some previous studies [9, 11, 14]. Interestingly, the results using multiple logistic regressions indicated that BMI, WC and WHtR were closely related to the incidence of dyslipidemia in femals centenarians, including high TG, high LDL-C and low HDL-C. This suggests that body measurement indices are important non-invasive indicators in evaluating health, which is used to assess lipid metabolism abnormalities. Unfortunately, due to the insufficient number of male centenarians, we failed to complete the multiple logistic regression analysis. We still need to continue our investigation and preserve these precious data to further guide our research.

## Conclusion

Hainan centenarians were short in stature and underweight. Moreover, female centenarians were often pear-shaped, while male centenarians were often apple-shaped. Further, BMI, WC and WHtR were well-correlated with the serum lipid, and TC, TG, LDL-C and HDL-C were significantly higher in females than males. Also, BMI, WC and WHtR were closely related to the incidence of dyslipidemia in females, including high TG, high LDL-C and low HDL-C.

## Abbreviations

BMI: Body mass index
CVD: Cardiovascular disease
DBP: Diastolic blood pressure
HC: Hip circumference
HDL-C: High-density lipoprotein cholesterol
LDL-C: Low-density lipoprotein cholesterol
SBP: Systolic blood pressure
TC: Total cholesterol
TG: Triglyceride
WC: Waist circumference
WHR: Waist-hip ratio
WHtR: Waist-to-height ratio
